# Away from the herd: loneliness as a dysfunction of social alignment

**DOI:** 10.1093/scan/nsae005

**Published:** 2024-01-27

**Authors:** Simone G Shamay-Tsoory, Alisa Kanterman

**Affiliations:** Department of Psychology, University of Haifa, Haifa 3498838, Israel; Department of Psychology, University of Haifa, Haifa 3498838, Israel

**Keywords:** herding, loneliness, social alignment, synchrony, social brain

## Abstract

The tendency of all humans to experience loneliness at some point in their lives implies that it serves an adaptive function. Building on biological theories of herding in animals, according to which collective movement emerges from local interactions that are based on principles of attraction, repulsion and alignment, we propose an approach that synthesizes these principles with theories of loneliness in humans. We present here the ‘herding model of loneliness’ that extends these principles into the psychological domain. We hold that these principles serve as basic building blocks of human interactions and propose that distorted attraction and repulsion tendencies may lead to inability to align properly with others, which may be a core component in loneliness emergence and perpetuation. We describe a neural model of herding in humans and suggest that loneliness may be associated with altered interactions between the gap/error detection, reward signaling, threat and observation-execution systems. The proposed model offers a framework to predict the behavior of lonely individuals and thus may inform intervention designs for reducing loneliness intensity.

## Introduction

The marvel that makes tens, hundreds and even thousands of separate organisms gather into an assembly that takes a life of its own is observed in numerous life forms including herding sheep, swarming ants, flocking birds and shoaling fish (Strömbom *et al*., 2014). Human crowds show similar manifestations of herding during various behaviors, including collective dance (Lee *et al*., 2020), marching (Wiltermuth and Heath, 2009), traffic (Helbing, 2004), joint clapping and synchronized steps when walking (Bernieri and Rosenthal, 1991; Burgoon *et al*., 1995; Schmidt and Richardson, 2008). Herding may be displayed also in ‘higher level’ emotional and cognitive forms, when emotions are communicated and propagate in large groups (Del Vicario *et al*., 2016) or when beliefs and fashion preferences spread within cultures (Bikhchandani *et al*., 1992).

The various displays of herding in humans indicate that a basic herding instinct drives humans to form connections and groups (Shamay-Tsoory *et al*., 2019). Indeed, among the most striking features of human behavior is the relentless search for social connections. From early age, we develop familial bonds and create multiple friendships with peers. In adolescence, these friendships become our main social focus and the search for romantic bonds intensifies. Throughout adulthood, we are driven to continue to create and maintain significant interpersonal relationships, which need to be of at least minimal in quantity and quality (Baumeister and Leary, 2017).

When the need to connect with others is not satisfied, people may experience loneliness. Loneliness is the subjective perception of social isolation, which is related but not identical to objective social isolation (Perlman and Peplau, 1981). Loneliness is also different from positive solitude, which is defined as isolation by choice that has various benefits (Long and Averill, 2003). Experiencing loneliness is common and can vary throughout the week or even within a single day, depending on social situations and events (1990). It also fluctuates throughout the lifespan with increased risk in older adulthood (Dahlberg *et al*., 2022) and adolescence (Von Soest *et al*., 2020).

Loneliness can be situational (acute and transient) or chronic (prolonged and ‘trait-like’). While situational episodes of loneliness are experienced by almost all humans in situations of objective isolation (e.g. moving to a different country, being excluded by others) or loss (De Jong-Gierveld and Raadschelders, 1982), chronic loneliness is a more stable state. Situational loneliness may arise in specific situations with specific interaction partners and may reduce in a later interaction, while chronic loneliness occurs on larger time scales (at least for 2 weeks and for most of the day), perhaps after repeated and accumulating interactions that involved situational loneliness, and is more difficult to alleviate (Shiovitz-Ezra and Ayalon, 2010). Both situational and chronic loneliness are associated with adverse consequences for well-being, physical and mental health and mortality (Leigh-Hunt *et al*., 2017; Rico-Uribe *et al*., 2018), although mortality rates in chronic compared to situational loneliness are higher (Shiovitz-Ezra and Ayalon, 2010). Notably, previous research on chronic loneliness as well as recent evidence related to the effects of social distancing during the Coronavirus disease 2019 (COVID-19) pandemic suggests that loneliness is a risk factor for the development of many psychiatric disorders including anxiety, depression and psychosis (Allé and Berntsen, 2021; Okruszek *et al*., 2020). Accordingly, there is a growing need to gain new insights into the underlying mechanisms of loneliness in order to develop more effective treatment designs.

Considering that loneliness represents an insufficient sense of connectedness with others, we propose here an approach that synthesizes models of herding with theories of loneliness. To this end, we apply a biological model according to which schooling, flocking and swarming are based on principles of attraction, repulsion and alignment and show how these principles offer a new understanding of loneliness in humans. We aim to support this approach by proposing an interdisciplinary hypothesis of the lonely brain and demonstrate that loneliness can be explained as an evolved biobehavioral strategy that responds adaptively to misalignment.

Toward these ends, the review has three parts. In the first, we discuss the herding principles that include attraction, repulsion and alignment in the animal kingdom and how they relate to human social alignment. The second part discusses how these principles are related to loneliness and its behavioral manifestations. Finally, the third part draws on literature regarding the neural basis of approach/avoidance and alignment in humans to posit a neural model of loneliness. The ‘herding model of loneliness’ presents a new approach to understanding loneliness and may serve as a roadmap for future interventions.

### Herding in the animal kingdom

While there are some detriments to sociality, including stress related to competition and increased risk for infectious diseases (Young *et al*., 2014; Majolo *et al*., 2016), herding has many benefits including better access to resources such as food (Reebs, 2000; Benoit-Bird and Au, 2009) and mating opportunities (Bowyer *et al*., 2020). Being connected to the group also improves survival by enabling predator confusion and improving the detection of predators via the coordination of responses (the ‘many eyes hypothesis’) (Olson *et al*., 2015). Furthermore, in the presence of threat, herds aggregate by moving toward the center and evade their predators, and survival rates are lower for marginalized animals (‘selfish-herd theory’, Quinn and Cresswell, 2006; King *et al*., 2012).

**Fig. 1.**
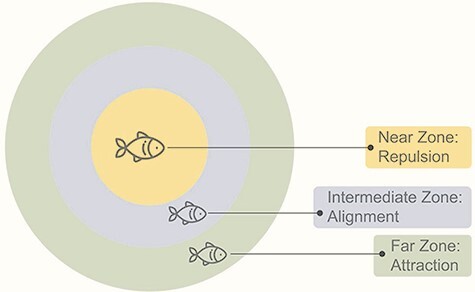
The attraction-repulsion theory. In the zone of attraction (far zone), the individual is driven to get closer to its neighbors and avoid being on the periphery. In the zone of repulsion (near zone), the individual is repelled away from others to avoid collision. The zone of alignment (intermidiate zone) represents aligning in the direction which allows collective movement by attempting to be in the same direction as the near neighbor. An individual is repelled from neighbors in the near zone, aligns the velocity with neighbors in the intermediate zone, and is attracted to neighbors in the far zone.

Beyond the clear benefits of herding, the regularities and similarities in herding activities between various species have incited biologists to identify the central tenets that govern the emergence of this behavior. It was suggested that in groups of animals involving multiple members, herding relies on local interactions between group members that are characterized by simple elements (Kelso, 1995; Camazine *et al*., 1999; Conradt *et al*., 2009; Conradt and List, 2009; Goldstone and Gureckis, 2009). The central theory describing these principles is the ‘attraction-repulsion’ framework that assumes three basic rules: attraction, repulsion and alignment. These principles apply differentially in three zones surrounding the animal: the far zone, the near zone and the intermediate zone (Figure 1). The rule of ‘attraction’ applies to the far zone and indicates that when the animal is too far from its neighbors, it moves toward them to ensure group cohesion. The rule of ‘repulsion’ applies to the near zone and indicates that the animal should move away from neighbors if they are too close, to avoid collisions. Finally, the rule of ‘alignment’ applies to the intermediate zone and indicates that the animal should match its direction with the direction of its neighbors, to generate a common motion of the entire group.

While the attraction-repulsion theory was derived from early research on fish schooling (Vine, 1971; Couzin, 2009; Schellinck and White, 2011), the power of this approach, which is based on physical principles, is that it enables capturing the core laws of collective motion in various life forms (Weitz *et al*., 2012). The attraction-repulsion theory was further supported by experimental setups that include three-dimensional motion tracking systems in both humans and animals (Wolinski *et al*., 2014), as well as with computer-based simulations (Vicsek and Zafeiris, 2012). The same principles have been also reported in the visual domain (Dachner *et al*., 2022) and in the movement of humans in a computerized paradigm (Belz *et al*., 2013). For example, Marton-Alper *et al*. (2020) have recently showed that in a computer game involving four human participants represented as colored circles moving on the screen, spontaneous synchrony—defined as interpersonal alignment in time and direction that occurs without conscious intention (Zamm *et al*., 2016)—can be detected based on the measurements of synchrony in movement (alignment), level of cohesion (attraction) and separation between circles (repulsion). Note that synchrony is a form of alignment, which also includes the component of temporality, in addition to directionality, and that alignment is dependent on successful attraction-repulsion regulation. These findings indicate that the movement of humans in groups may be governed by the same basic components of herding described in group-living animals.

In the context of herding in humans, here we define herding as the behavior that sustains humans in large groups, by keeping cohesion as a collective based on local interactions. Local interactions occur between individuals who are close in proximity (physically but also emotionally and cognitively) and are carried out via processes of attraction (getting near/closer to individuals of interest, to not disperse), repulsion (getting away from others to not collide with them) and alignment (directional synchrony, i.e. two or more individuals are moving in the same direction or thinking similar thoughts, at the same time), similarly on all levels of herding. We hold that herding in humans is dynamic as collisions occur, and individuals disperse, thus denying synchrony temporarily. However, repulsion following collision may allow for individuals to attract and realign when comfortable distance is achieved.

### Different levels of herding in humans

**Fig. 2.**
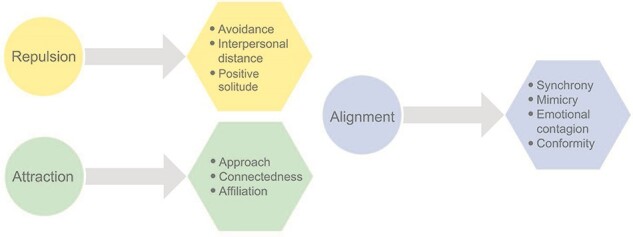
Different levels of attraction, repulsion and alignment. The principles of attraction, repulsion and alignment share many similarities with several psychological concepts including approach, avoidance and synchronization. Repulsion and attraction can be expressed through physical distance or closeness, respectively, but also through psychological distance. Affiliation refers to the sense of belonging or association with a particular group or groups that may increase when interpersonal synchrony is high (Leary, 2010). Synchrony, on the other hand, refers to the alignment in time and space between two or more individuals, in movement, emotion or cognition.

While the herding principles may explain how collective movement emerges in large groups, the question remains whether and how these basic rules can offer a framework for understanding ‘higher’ levels of herding in humans, such as in the case of emotional and cognitive forms of alignment (Figure 2). According to the model, repulsion and attraction can be expressed through physical distance or closeness, respectively, but also through psychological distance such as in the case of moral values. Moreover, we propose that repulsion may also include processes of conscious disengagement and positive solitude. Disengaging from others allows to calibrate distance and avoid collision either physically, emotionally or cognitively and therefore allows for later reconnection.

Positive solitude, a state that arises when an individual consciously chooses to spend time alone (Ost Mor *et al*., 2021), allows regulating social distance not only from individuals one does not want to affiliate with, but also from familiar and close others, when needed. This form of voluntary isolation is deemed beneficial for mental well-being as it grants freedom from societal pressures (Lay *et al*., 2019), encourages self-exploration (Long and Averill, 2003) and facilitates emotional self-regulation (Nguyen *et al*., 2018). Accordingly, recent studies focused on ‘aloneliness’, defined as the negative psychological state that arises from a dissatisfaction from one’s lack of solitude (Swets and Cox, 2022), further emphasizing the need to be alone at times (positive solitude) (Leary *et al*., 2003). It is therefore possible that positive solitude is regulated by the repulsion system, as it is driven by exaggerated closeness and drives individuals to calibrate their distance from others. Hence, as recently suggested, flexibility in synchrony may be crucial for social interaction (Mayo and Gordon, 2020), i.e. getting in and out of synchrony in a dynamic manner.

Notably, the principles of attraction and repulsion share many similarities with early theories of approach-avoidance in humans. These theories consider the processes of approach and avoidance as basic elements to describe human behavior in terms of sensitivities and responses to appetitive and aversive stimuli (Gray, 1981). Distinguishing approach from avoidance was addressed by numerous philosophers and commentators across disciplines. The early work of Lewin (1935) viewed approach as the energization of behavior toward a stimulus, whereas avoidance was described as representing the energization of behavior away from stimuli. Recent distinctions between approach and avoidance refer to differences in how people react to life events and are a result of differences in affective, cognitive and behavioral response patterns, which are relevant to adaptive functioning (Elliot and Church, 1997; Heller *et al*., 2007).

Importantly, the concepts of approach and avoidance were presented as explaining not only physical approach toward concrete objects but also cognitive approach toward ideas, views and people. In line with this idea, classical studies on approach and avoidance show that physical and abstract forms of approach and avoidance are interrelated. For example, it was found that participants are faster to physically pull a joystick than to push it away in response to positive words, while in response to negative words, they are faster to physically push the joystick away than to pull it toward themselves (Chen and Bargh, 1999). Likewise, it was shown that interpersonal space, defined as the physical distance maintained between people, is associated with higher levels of emotional connectedness, such that people prefer to stand closer to people they like (Scheele *et al*., 2012; Cohen *et al*., 2017).

While attraction/repulsion is postulated to regulate distance similarly to approach/avoidance, the herding model also includes the critical component of alignment and suggests that the attraction/repulsion zones serve to allow individuals to synchronize with each other dynamically. Thus, the model advocates that in addition to approach/attraction and avoidance/repulsion, synchronization (a form of alignment) is an additional fundamental building block of social behavior, which underlies the sense of connection. In particular, the model emphasizes the interconnectedness of the three components and the dependency of synchrony on distance and shows how they may be orchestrated together and shape social behavior.

The idea that physical dimensions of behavior are interdependent with emotional and cognitive dimensions is in line with the ‘embodied cognition’ theory, according to which bodily states affect cognition, and cognitive states affect the body (e.g. Niedenthal *et al*., 2005). Similarly, the construal level theory of Lieberman *et al*. (2007) proposes the concept of ‘psychological distance’, according to which different distance dimensions (time, space and social) are interrelated, in that they are associated with each other, affect each other and add to each other to produce a unified sense of distance. Psychological distance refers to a perceived rather than objective distance between oneself and other individuals, as well as from objects, events or abstract concepts and phenomena (in space or time), that in turn influence the individuals’ thoughts, emotions and actions (Liberman and Trope, 2014). The concept of psychological distance may help explain how the physical levels of herding are also experienced emotionally and cognitively, as it refers to the level of connection that individuals feel with others or the group. In line with this, the embodiment theory, or embodied cognition, suggests that cognitive and emotional processes are influenced by the body, such that perception and motor action planning are at the basis of higher functions, and are not restricted only to the brain (Janzen, 2022; Gallagher, 2023). Research has shown that bodily states influence how people understand others and interact with them (Hardy, 2021). Thus, physical distance or proximity may influence the perception of psychological distance, and physical misalignment may elicit psychological misalignment and vice versa.

Evidence suggests that all three levels of herding (emotion, cognition and movement) that influence connection may be disrupted in loneliness: loneliness is negatively correlated with positive emotional contagion (joy and love) (Borawski *et al*., 2021) and poor motor synchronization skills (Saporta *et al*., 2023). Furthermore, the social expectation that people should be ‘happy’ under certain conditions (such as during a pleasant social interaction) also leads to increased feelings of loneliness when this expectation is not met (Bastian *et al*., 2015). Finally, high loneliness was recently associated with greater perceived cognitive distance between themselves and their friends (Kokici *et al*., 2021).

It is therefore possible that the principles of attraction and repulsion found in animal herding are antecedents of more abstract levels of social approach and avoidance in humans. In a similar manner, the principle of alignment in movement direction may represent an antecedent for higher level of alignment achieved via more complex forms of information transfer such as verbal communication (Conradt and Roper, 2005; Conradt and List, 2009; Fichtel *et al*., 2011; Boos *et al*., 2014). Indeed, social alignment also seems to be displayed at the emotional level, when emotional states are transferred in groups, such as in the case of emotional contagion in person and via social media (Kramer *et al*., 2014). Social media users tend to attract like-minded people and repulse from dissimilar others, reinforcing a shared narrative and bias the information diffusion between users, thus increasing alignment and cohesion between them (echo chambers) (Cinelli *et al*., 2021). Rapid transfer of information can also trigger the propagation of beliefs and attitudes, and group deliberation can even lead to conformity in views and moral conventions (Greenwood *et al*., 2011; Marton-Alper *et al*., 2022). Likewise, it has been proposed that movement synchronization is linked to emotional synchronization such that alignment in motion predicts quality of emotional relationship (Ramseyer and Tschacher, 2011).

While the exact division to physical zones is not fully applicable to more abstract levels of herding, the attraction-repulsion-alignment principles may operate as general organizing principles of how we interact with others. Therefore, assessing herding behaviors in humans may foster the understanding of conditions in which individuals fail to connect with others, objectively or subjectively, such as in the case of isolation and loneliness. According to the herding model, the basis of physical and emotional connection is the ability to synchronize on all levels: emotional, cognitive and in movement. The ability to synchronize is dependent on proper regulation of social distance. Thus, well-regulated attraction, repulsion and therefore alignment are all essential for adaptive social functioning; however, they can become dysfunctional in cases of loneliness. This dysregulation may arise from or be related to abnormal activity in systems associated with gap detection, reward processing, repulsion or observation execution.

### Attraction, repulsion and alignment as characteristics of loneliness

Evolutionary theories hold that loneliness is a common human experience, and similarly to hunger, which evolved to signal that a person needs to consume food to maintain energy levels, it evolved as a signal to reconnect with others to maintain social bonds (Cacioppo *et al*., 2014). Conversely, evolutionary accounts to loneliness also assert that when loneliness is chronic, social interactions may become threatening and lead to increased withdrawal behaviors (Cacioppo and Cacioppo, 2018). These two contrasting forces of social approach and avoidance seem to be at the heart of the distress experienced by lonely individuals, positioning them in an intractable conflict between the need to connect and the need to avoid social threat (Hawkley and Cacioppo, 2010).

Early studies on approach and avoidance motivation in loneliness generally demonstrated that high approach motivation is associated with less loneliness, whereas avoidance motivation is associated with higher levels of loneliness (Gable, 2006). In line with this, in a recent study, loneliness was associated with reduced representational similarity between the self and others (Courtney and Meyer, 2020), including their friends (Kokici *et al*., 2021). Approach and avoidance motivation in lonely individuals were also measured physically by preferred interpersonal distance. Layden *et al*. (2018) showed that chronic loneliness was associated with greater interpersonal distance within the intimate space but not within the relational (family and friends) or collective spaces (acquaintances). Lieberz *et al*. (2021) showed that lonely individuals prefer greater physical interpersonal distance from unfamiliar people (strangers). However, Saporta *et al*. (2021) showed that while chronic loneliness is associated with overall greater preferred interpersonal distance, COVID-19-related loneliness, a situational loneliness, was associated with smaller preferred distance across conditions (Saporta *et al*., 2021). This study may therefore indicate that situational loneliness may drive people to approach others, whereas chronic loneliness may rather increase avoidance motivation.

Notably, Lucas *et al*. (2010) showed that when lonely individuals are presented with acceptance cues in the form of vignettes depicting safe and including social environment, they exhibit higher social motivation during a social interaction, such as increased mimicry, a signal of social affiliation (Lucas *et al*., 2010). Moreover, lonely individuals show higher motivation for inclusion in a ball tossing game when low but not high effort is required in order to be a part of a group interaction (Kanterman *et al*., 2022). Finally, Smith and Pollak (2022) showed that high resting parasympathetic activity (a physiological marker associated with flexible adaptation) in lonely individuals predicted increased social approach behavior, while low activity predicted increased social avoidance behavior, further indicating that there is an important link between loneliness and approach-avoidance which influences social behavior.

Overall, these findings demonstrate that lonely individuals may fluctuate between hyper-approach and hyper-avoidance, depending on the context (interacting with familiar *vs* unfamiliar others, for example). It thus seems that while these two forces play a key role in the social behavior of lonely individuals, the link between approach-avoidance and loneliness depends on the type of loneliness (situational or chronic) and social context (safe *vs* threatening). Moreover, in line with the herding model, as alignment depends on the optimal distance regulated by the attraction and repulsion zones in animals, synchronization in humans may similarly depend on approach and avoidance behaviors. This view suggests that loneliness is a dynamic emotional state that can influence individuals’ motivation and behavior, depending on the levels of loneliness. Thus, hyper- or hypo-approach/avoidance may directly affect the ability to align and synchronize with others not only in movement but also in emotion and cognition and thus contribute to loneliness emergence and severity in humans.

Interpersonal synchrony is a key factor in creating positive interactions, promoting affiliation and feelings of connection (Marsh *et al*., 2009; Vacharkulksemsuk and Fredrickson, 2012; Rabinowitch *et al*., 2015). Numerous studies support the idea that synchrony enhances this sense of connection. Social connectedness is broadly defined as the person’s subjective awareness of being in close relationships with others (Lee and Robbins, 1995) and refers to the bonds between individuals, unrelated to a specific group (James *et al*., 2017). Physical synchronization, for example, is related to emotional connection (Ramseyer and Tschacher, 2011). It has been suggested that affective synchrony (the embodiment of perceived emotions) allows for better information exchange between individuals and interpersonal (as opposed to individual) emotion regulation and therefore contributes to stronger social bonds (Wood *et al*., 2021). Finally, in mother–child dyads, synchrony has been associated with higher shared affect, smiles and mutual gaze, effective turn-taking and harmonious interactions, indicating that synchrony is a key factor in human interaction (Leclère *et al*., 2014). Thus, synchrony is a specific mechanism that contributes to the establishment and the sustainment of connection throughout time. It is therefore possible that loneliness is associated with poor synchronization skills.

Saporta *et al*. (2023) have recently found that lonely individuals show reduced ability to adapt their movements to those of their partner and synchronize while exhibiting increased activation in the observation-execution system, possibly representing increased neural effort when trying to align with others. Evidence suggests that this process also happens when decoding other people’s emotions and beliefs (Jabbi *et al*., 2008; Rizzolatti and Sinigaglia, 2016) and is not restricted only to motor actions. Heerdink *et al*. (2013), for example, showed that individuals who feel rejected tend to seek social acceptance by exhibiting heightened conformity, which can be viewed as cognitive alignment. Related to synchrony is mimicry, the process of copying the actions of another, which contributes to social bonding as well (Palagi *et al*., 2020). Facial mimicry, in particular, tends to elicit emotional contagion between individuals (Winkielman *et al*., 2016). Interestingly, positive emotion contagion was found to be a negative predictor of loneliness (Borawski *et al*., 2021). Lakin and Chartrand (2005) found that situationally excluded individuals mimic strangers more, presumably due to the enhanced motivation to create new connections. Chronic loneliness, on the other hand, was associated with impaired spontaneous smile mimicry (smile back less), while deliberate mimicry was intact (Arnold and Winkielman, 2021).

Collectively, these findings provide indication that loneliness may entail dysregulation of all components of herding including attraction, repulsion and alignment: lonely individuals have difficulties in approaching and aligning with others at all levels, from movement alignment to cognitive and emotional forms of alignment. Dysregulated social behavior may be manifested in either diminished or exaggerated approach, avoidance or synchronization behaviors. Broadly, it is possible that in situational loneliness approach motivation increases, driving lonely individuals toward reconnection and hyper-alignment. However, in chronic loneliness, which is considered as a stable state once developed, avoidance motivation and hypo-alignment increase instead, causing difficulties in attuning and connecting with others. In order to align with others and feel connected, individuals need to be able to maintain an optimal distance from conspecifics, that is neither too far nor too close. It is thus possible that herding animals as well as humans monitor their distance from others at all times, and once a gap is detected (in distance or direction), behaviors that aim at minimizing that gap come into play. We therefore propose that herding in humans is driven by neural mechanisms that are associated with gap monitoring and detection, approach and avoidance behaviors and reward.

## Brain networks that may support herding and their relevance to loneliness

Research examining the neural underpinnings of herding has generally looked at how the brain responds to different forms of alignment, ranging from motor synchrony to emotional contagion and social conformity. Recent models of social alignment hold that herding entails the interaction between a gap detection system, which includes the dorsal anterior cingulate cortex (dACC) and the anterior insula (AI), supporting the evaluation of the distance between the individual and others; a reward circuitry including the ventral striatum (VS) and the orbitofrontal cortex (OFC), signaling connectedness; and the observation-execution system, which includes the inferior frontal gyrus (IFG), inferior parietal lobule (IPL) and the premotor cortex and possibly supports alignment with the direction and velocity of other individuals in the group (Shamay-Tsoory *et al*., 2019). The multifaceted role of different brain regions and networks is highly complex; hence, we use a reductive model in which we focus on a small number of regions representing the different networks which may be most relevant to herding behavior based on correlative neuroimaging studies. However, we acknowledge that future research focusing on the empirical examination of the theory and the involvement of the aforementioned regions is required.

**Fig. 3.**
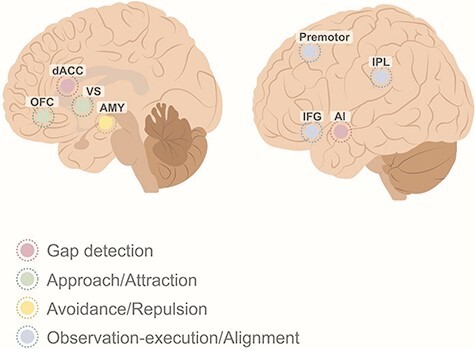
Neural networks associated with loneliness. The neural mechanism underlying herding behavior in terms of attraction, repulsion and alignment. AMY: amygdala; premotor: premotor cortex.

Notably, regions in the gap detection, reward circuitry and the observation-execution system were found to show anatomical and functional abnormalities in loneliness. In addition to these systems, the amygdala, a core component of avoidance and repulsion, was also repeatedly implicated in loneliness. Indeed, a recent coordinate-based meta-analysis of neuroimaging studies revealed that functional clusters across the striatum, IFG and AI converged to be significantly related to loneliness (Wong *et al*., 2022). Similar regions were reported in a review of structural and functional studies of loneliness (Lam *et al*., 2021), pointing to the involvement of the AI, amygdala and the VS. In addition, studies have found that the white-matter tracts involving the IFG, IPL (Nakagawa *et al*., 2015), temporoparietal junction and the AI predict the level of loneliness (Kanai *et al*., 2012; Tian *et al*., 2014). Thus, the four systems that likely support herding are also implicated in loneliness (Figure 3). While correlative, these studies provide initial support to the notion that loneliness is related to brain activity of regions that are strongly implicated in social behavior, and approach, avoidance and alignment in particular, although we acknowledge that they take part in other cognitive processes, such as in attention (Shulman *et al*., 2010).

### The gap monitoring system in loneliness: dACC and AI

Considering that loneliness is defined as a form of perceived discrepancy between the desired and actual social connections (Cacioppo and Cacioppo, 2018), feeling lonely can be conceptualized as experiencing a constant state of perceived gap between the self and others. The joint activity of the ACC and the AI was suggested to constitute a network calculating the extent of gap existing between internal and external input which provides a fundamental basis for evaluating self- and other-related differences and may therefore be relevant here (Craig, 2009). As the insula integrates upcoming visceroautonomic feedback and the ACC generates visceral responses, among other functions, these regions are believed to be parts of the salience network, a collection of regions playing a key role in processing ‘homeostatic relevance’, including in social context (Seeley, 2019). Indeed, increased activity in the salience network is frequently observed in situations in which it may be important to change behavior (Dosenbach *et al*., 2007) and is correlated with error detection (Ham *et al*., 2013). The dACC and AI are known to be implicated in encoding negative emotions (Corradi-Dell’Acqua *et al*., 2016) as well as monitoring errors (Ridderinkhof *et al*., 2004; Garrison *et al*., 2013). Yet, further investigation is required to determine whether these regions and networks are involved in herding behavior directly.

Numerous studies on social exclusion, which may be viewed as situational loneliness, have reported increased activation in the ACC and AI while being excluded (Eisenberger, 2012), indicating that the activity in the salience network is associated with the distress related to gaps between the self and the group, i.e. in a social context. Likewise, previous neuroimaging studies have reported activity in the ACC and AI in the processing of prediction error signals in paradigms involving social conformity, when there is a conflict between the views of the participant and the group (e.g. Levorsen *et al*., 2021). Activity in the ACC was also found in studies examining misalignment in motion (Cacioppo *et al*., 2014), indicating that the salience network may process the self-other gaps at all levels, from motion to emotion to cognition. In the context of herding, while still needs to be tested, it is thus possible that the gap detection system evaluates if the individual is too close or too far from others to calibrate the amount of distance needed to achieve alignment at all levels (motor, emotional and cognition). It is important to note that we do not claim that individuals must be aligned with others on all levels at all times in order not to feel lonely. However, if individuals are misaligned with others on one or more levels, they may experience loneliness, which in turn may further diminish their ability to reconnect. However, additional research is required to confirm or refute these hypotheses.

Poor evaluation of the gap between themselves and the group as too large or too small could underlie the proposed dysregulation of the repulsion and attraction tendencies in loneliness. The insula, which plays a significant role in affect, empathy (Singer *et al*., 2009) and emotional awareness via interoceptive representations (Craig, 2009), has been implicated in many studies on loneliness when studies in a social context. For example, individuals with selective lesions to the right AI are less likely to report loneliness in comparison with controls (Cristofori *et al*., 2019). However, results regarding insula responsiveness in loneliness are mixed. Studies show increased activation of the AI during the processing of positive social stimuli (compared to positive non-social stimuli) among lonely individuals, while non-lonely individuals show greater VS activity in the same condition (Cacioppo and Hawkley, 2009). In schizophrenia, AI activation is positively correlated specifically with the severity of loneliness (Lindner *et al*., 2014). Interestingly, Lieberz *et al*. (2021) found that lonely individuals exhibit blunted functional connectivity between the AI and occipitoparietal regions during trust decisions, and Morr *et al*. (2021) found that loneliness is correlated with diminished reactivity in the AI as well as the ACC during exposure to emotional facial expressions. Wong *et al*. (2022) suggest that blunted neural activity related to affective processing in loneliness may be elicited due to increased cognitive control in the purpose of enhancing affective regulation. This, with time, may cause system exhaustion and affective dysregulation.

The herding model of loneliness suggests that loneliness is an adaptive response to misalignment, which begins with the detection of the gap—perceiving oneself as disconnected from the group—and gives rise to processes that promote regaining alignment, specifically adjusting the distance from the group in a way that will allow optimal alignment, i.e. not too far nor too close. It is therefore possible that dysregulated gap detection may alter the attraction-repulsion-alignment systems, causing or related to abnormalities in all the components of herding behavior, thus aggravating loneliness even further. Distorted gap monitoring may manifest as not detecting a gap when there is one due to emotional hypo-sensitivity, detecting a gap when there is none or perceiving the gap as larger or smaller than it is due to cognitive biases. However, more empirical research is needed regarding gap detection distortions in the context of loneliness.

### Attraction in loneliness: the role of the reward circuitry (VS and OFC)

Studies show that lonely individuals exhibit an abnormal response to positive social stimuli such that they report less positive affect and show diminished responsiveness in the reward system (Lam *et al*., 2021). The neurobiology of attraction in humans is based on studies of approach motivation, which is thought to be mediated primarily by the reward circuitry. This circuitry includes the mesolimbic dopamine projections from the ventral tegmental area (VTA) to the VS and the mesocortical dopaminergic projections from the VTA to the prefrontal cortex including the OFC (Knutson and Cooper, 2005). Evidence from neuroimaging studies supports the link between social attraction and reward by demonstrating increased activity in the reward circuitry during interactions with loved ones and with cooperative partners (Vrtička and Vuilleumier, 2012). Similarly, the VS and the OFC, among others, have been shown to be activated when participants approach and align their opinions with that of the group (Minich *et al*., 2023). It is therefore not implausible to assume that these regions also play a part in the attraction system of the proposed herding model.

Studies on the reward system in loneliness show reduced VS activation when presented with pleasant social images, compared to non-lonely individuals (Cacioppo *et al*., 2009). However, another study revealed that higher levels of loneliness actually predicted greater VS activity in response to seeing close others compared to strangers, while at lower levels of loneliness there was no such difference (Inagaki *et al*., 2016). The proposed interpretation of this finding was that lonely individuals do not view strangers as possible targets of social connection, and therefore, they focus their ‘social craving’ on people they already know. Another possible interpretation is that lonely individuals are not interested in satisfying their social needs through strangers. More recently, however, a functional imaging study of younger and older adults reported no association between loneliness and VS activation in response to pleasant social images of strangers in either age group (D’Agostino *et al*., 2019), indicating that abnormalities in the VS are not always present in loneliness, and that the VS is insufficient in explaining alterations in reward responses. Structurally, Sin *et al*. (2018) found an association between loneliness and gray matter volume in the striatum among older individuals with depression, such that participant with a single depressive episode showed a positive correlation between striatum volume and loneliness, while participants with multiple depressive episodes showed a negative correlation between striatum volume and loneliness, possibly pointing toward the possibility that the involvement of the VS is influenced by the presence or severity of psychiatric symptoms.

Social animals such as rats show increased social approach following social isolation (the only parallel to loneliness in animals), which is mediated by dopaminergic activity. However, isolation also increases food and drug intakes, suggesting broad changes in the reward system (Lallai *et al*., 2016). This increase can be reversed by exposing the animals to an hour-long daily interaction after being isolated (Raz and Berger, 2010), indicating that even short social exposure can alleviate the adverse effects of isolation. Acute social isolation in humans (10 h with no contact) increases craving and obliterates midbrain response for social contact (Tomova *et al*., 2020). Individuals with preexisting chronic loneliness, however, show reduced activation in the substantia nigra and the VTA when isolated, compared to individuals who are not considered as lonely prior to isolation. Interestingly, social rejection or exclusion elicits activation in the ACC but does not impact midbrain or reward-related regions, possibly indicating that there is a fundamental difference in reward activation in situational *vs* chronic loneliness (Tomova *et al*., 2021). In the latter study, the OFC, a core region in the reward system, was reported to show a stronger response to social cues following isolation, compared with fasting. Food cues after fasting, on the other hand, evoke stronger response in the amygdala and the ACC, but not in the OFC, pointing to the latter’s role specifically in social cognition (Tomova *et al*., 2020). However, as loneliness is associated with high substance use rates (Ingram *et al*., 2020), it can be argued that when connection cannot be achieved, individuals turn to other forms of rewarding stimuli as compensation, which may further increase loneliness and isolation and decrease the possibility for connection.

The OFC is further implicated in attraction as lesions in the OFC are associated with smaller preferred interpersonal distance, regardless of the target’s identity (Candini *et al*., 2020). The OFC has been indicated in the evaluation of negative as well as positive facial expressions, however more prominently for the former (Ruffman *et al*., 2008). This in turn possibly affects processes of social decision-making and causes inappropriate social behavior, such as approaching in an enhanced manner toward unfamiliar targets displaying negative emotions, as one study found (Willis *et al*., 2010). In mice, the OFC is the region that is affected the most following social isolation, as well as its projections to other regions (Liu *et al*., 2016).

In line with the herding model of loneliness, we propose that increased social approach may characterize mainly situational loneliness (such as in the case of social rejection), which is supposed to promote reconnection with others. However, in chronic loneliness, avoidance motivation may increase as a form of protection, and during social interactions, activation in the reward system may be reduced. In both cases, we propose that the ability to align with others may be hindered due to dysregulated interpersonal distance.

### The repulsion system in loneliness: amygdala

The evaluation of diminished gap between the individual and others may signal the violation of self-boundaries and trigger discomfort and avoidance (Regoeczi, 2008). Therefore, the brain systems activated during repulsion may rely on survival-enhancing tendencies of aversion, which operate through the activation of sympathetic fight-or-flight circuits in the limbic system including the amygdala (MacDonald and MacDonald, 2010). In addition to the amygdala, Vieira *et al*. (2020) showed that personal space intrusions, by social or non-social stimuli, recruit frontoparietal structures such as the ventral premotor cortex, the intraparietal sulcus and the midbrain periaqueductal gray. Intrusions made by social, compared to non-social stimuli, however, were related to increased functional connectivity between the midbrain and the premotor cortex. The authors interpreted these results as showing that when faced with intrusions, the brain recruits defense mechanisms (i.e. the instinct to move), which may be relevant to social intrusions as well.

In line with the role of the amygdala in repulsion, Kennedy *et al*. (2009) have found that the amygdala is differentially activated by proximity to another person, and that lesion in this region results in the absence of space boundaries. These findings were later replicated in various paradigms showing that the amygdala generally responds to the approaching of threatening stimuli (Mobbs *et al*., 2010; Coker-Appiah *et al*., 2013; Vieira *et al*., 2017). Interestingly, previous studies have found that the social network size of participants, which reflects objective isolation when low, predicts the level of amygdala activation (Bickart *et al*., 2011; Von Der Heide *et al*., 2014; note that this was not replicated by D’Agostino *et al*., 2019). This may indicate that a dysfunction in amygdala activity can affect the number of social relationships and vice versa.

In a study where participants were asked to move toward or away from emotional stimuli (positive, neutral and negative images), participants with high scores on volatility, which is associated with fight-or-flight responses, showed increased amygdala activation in response to negative but not positive stimuli whether they were approached or avoided. In contrast, participants with higher withdrawal scores, which are associated with behavioral inhibition, showed increased amygdala activation in response to all stimuli, regardless of valence (Cunningham *et al*., 2010). Furthermore, a study that used a machine learning approach found that the amygdala serves as a key node which contributes to the prediction of loneliness (Feng *et al*., 2019). These findings are in accordance with the role of the amygdala in social processing and interaction (Adolphs *et al*., 2005; Rutishauser *et al*., 2015).

Similarly, a recent voxel-based morphometry study reported that decreased gray matter in the amygdalo-hippocampal complex volume predicted levels of loneliness (Düzel *et al*., 2019). Tian *et al*. (2016) found a positive association between gray matter volume of the left amygdala and the extent of social distress, and that this association was mediated by loneliness. However, in older adults, loneliness was positively associated with lower gray matter volume in the same region (Düzel *et al*., 2019). In addition, in a study that involved an intervention to reduce loneliness in older adults, participants with larger amygdala volumes experienced greater reductions in loneliness. Moreover, Wong *et al*. (2016) found that loneliness was associated with a weaker connectivity between the amygdala and the superior frontal gyrus, which is associated with self-awareness and social behavior. Finally, Beadle *et al*. (2022) have recently reported that patients with amygdala damage had smaller social networks and higher loneliness scores compared to patients with hippocampal damage. However, amygdala activity depends on the context (Adolphs & Spezio, 2006) and is also altered in loneliness (Lam *et al*., 2021). We therefore would expect that individuals with high loneliness would exhibit hyper-activation in the presence of strangers and hypo-activation in the presence of close others or other social targets that are conveyed as safe.

These findings indicate that in the context of herding, the amygdala may mediate the repulsive force that helps to maintain an appropriate physical and psychological space surrounding the individual. As suggested earlier, it is possible that the direction of activity depends on the context and on whether loneliness is situational or chronic. In situational loneliness, the activation level of the amygdala is supposedly reduced in order to gain closeness, causing absence of space boundaries. However, in chronic loneliness, the amygdala may become hyper-activated due to the general stress that accompanies this condition, and lonely individuals may show increased avoidance motivation as a preferable strategy. In line with this, research shows that successfully avoiding an unwanted stimulus leads to a reward response in the brain, similarly to successful appetitive approach (Leknes *et al*., 2011). Further research is required, however, to determine whether amygdala hypo- or hyper-activity is evident in situational and chronic loneliness, respectively, and plays a role in herding.

### The observation-execution system and loneliness: IFG, IPL and premotor cortex

Following the regulation of the attraction and repulsion tendencies, the model proposes that the individual may engage in alignment in direction with the group. According to the model, difficulties in alignment may hinder the ability of lonely individuals to fully engage in social interactions and thus to connect. Studies on social alignment focus on synchrony, imitation, and mimicry functions, which are primarily attributed to the observation-execution system (mirror neurons). The observation-execution system is a network of brain regions reported to be activated both when individuals perform an action and when they observe others perform the same action (Rizzolatti and Sinigaglia, 2016). Because human studies investigating the observation-execution system are limited in their ability to demonstrate mirroring properties of specific neurons (Mukamel *et al*., 2010), studies use the term action-observation/observation-execution system, to denote the system of regions exhibiting similar activation levels during both processes.

The regions that are frequently included in this system in the human brain are the IFG, IPL and the premotor cortex (Molenberghs *et al*., 2012). The observation-execution system as a whole was shown to support adaptive online interaction (Cook *et al*., 2014). The IPL was shown to be active during both motor (Cacioppo *et al*., 2014) and emotional (Nummenmaa *et al*., 2008; Korisky *et al*., 2020) synchronization. Similarly, the IFG is activated during tasks involving finger tapping in synchrony (Fairhurst *et al*., 2013) and imitation (Belyk *et al*., 2016). Studies using hyper-scanning further demonstrate inter-brain coupling of the IFG in various types of tasks that involve synchrony between two or more individuals, including movement synchronization (Gamliel *et al*., 2021). We propose that this is the network that is most likely to govern synchronization.

Importantly, the IPL and the IFG have been shown to be involved in loneliness in structural and lesion studies (Tian *et al*., 2014; Nakagawa *et al*., 2015; Cristofori *et al*., 2019), as well as in functional studies (Cacioppo *et al*., 2009). For example, lesions to the right IFG have been associated directly with increased loneliness, indicating that this region is important in determining loneliness severity and is crucial for social functioning (Cristofori *et al*., 2019). In a recent study, Saporta *et al*. (2023) have shown that individuals with high-loneliness scores demonstrate diminished ability to synchronize their movement in a computer game, compared to low-loneliness individuals. Critically, while overall brain activity during synchronization periods was evident in the IFG and IPL, individuals with high-loneliness scores had higher activations in these regions compared to individuals with low-loneliness scores. Although correlative, these findings were interpreted as showing that lonely participants activate their observation-execution system more than low-lonely individuals when they attempt to synchronize. While the latter study focused on movement synchronization, similar findings were recently reported by Brilliant *et al*. (2022) who report increased functional connectivity between the IFG and the supplementary motor area during emotional interactions in high-lonely individuals, which although has a role in many cognitive functions, may represent here specifically the desire to reconnect with others. Hyper-activation of the observation-execution system may therefore be explained as representing an elevated urge to increase affiliation and liking between people. It remains to be seen, however, whether hyper-activation of this system is a consistent finding in loneliness in a social context and particularly in relation to herding behavior.

**Fig. 4.**
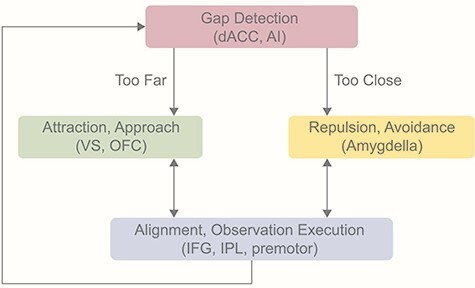
The loneliness cycle. The proposed neural mechanism for the herding model of loneliness. AMY: amygdala; premotor: premotor cortex. In relation to loneliness, we postulate that due to disturbances in the repulsion and attraction system, and perhaps in the gap detection too, individuals with high loneliness are unable to reach a state of alignment and therefore sense of connection, as their interpersonal distance is poorly regulated.

It thus seems that a possible key element of the difficulty that lonely individuals have to fully connect in social interactions may be related to impaired capacity to synchronize. These individuals, perhaps, may need to put more effort into aligning their behavior with others, which may be highly distressing. The hyper-activation of the observation-execution system reported in high-loneliness individuals may be explained by the need to compensate for their alignment difficulties. Importantly, this difficulty may stem from dysregulated attraction and repulsion tendencies in loneliness, which hinders proper alignment (Figure 4). Cacioppo *et al*. (2014) conceptualized loneliness as a self-sustaining cycle. Here, we suggest that this cycle is related to the three principles of herding and how loneliness may develop when viewed through this lens. While the two models share similarities and are viewed as regulatory mechanisms, the herding model of loneliness is unique due to the inclusion of the alignment component, which may explain the inability to regain sufficient connection, while still accounting for phenomena such as positive solitude.

Collectively, it is plausible to hypothesize that gap detection, reward, repulsion and observation-execution are possibly important components in the development of loneliness. The gap detection system may support the evaluation of the distance between the individual and the group. If the individual and others are too close, a repulsion/avoidance system may be activated to increase the social distance. However, if the distance is too large, an attraction/approach system, which may be supported by the reward circuitry, is activated to bring the individual closer and prevent disconnection. Finally, both the attraction and repulsion systems may regulate the activation of the observation-execution system and support alignment with the direction and velocity of other individuals in the group. Loneliness may entail dysfunction at each component, from having distorted gap monitoring to increased or decreased attraction, avoidance and alignment. The direction of hyper- or hypo-activations of these systems may depend on the context. For example, situational loneliness may increase approach and hyper-alignment, whereas chronic loneliness may increase avoidance and hypo-alignment. Nonetheless, dysregulation of herding behavior is expected in both situational and chronic loneliness.

## Conclusions and future directions

Here, we sought to contribute to the rapidly growing literature on loneliness and argue that loneliness may represent a disorder of herding. By extending previous applications of the biological attraction-repulsion-alignment theory of herding, we argue that these basic rules may serve as organizing principles of social interaction and may therefore also apply to emotional and cognitive forms of alignment in humans. While in animals three principles are described as strictly physical, in humans these principles may direct behavior at both physical and psychological levels. Specifically, we argue that the three classes of motives or actions are the fundamental building blocks of human social behavior, and when dysregulated, they may contribute to the development of loneliness. According to this model, well-regulated attraction, repulsion and alignment are all crucial for adaptive social functioning but may be dysfunctional in loneliness, which may be related to abnormal activity in the gap detection, reward, repulsion and/or observation execution systems.

Pending further empirical validation, we postulate that all three components and their underlying systems need to be interrelated and balanced. However, flexibility is important and calibration between systems is required. We therefore speculate that the ideal balance between the subcomponents of the herding model is related to synchrony flexibility—the ability to go in and out of synchrony with other social targets during an interaction and throughout relationships in general, thus dynamically adjusting to different social contexts. Flexibility in interpersonal synchrony is considered adaptive, as it allows the shifting from independence to synchrony when needed (Mayo and Gordon, 2020). However, as the model is still theoretical, it remains to be seen in future studies whether an optimal balance between the subcomponents can be detected.

While several brain networks were found to be relevant to loneliness, our model adds to the literature on loneliness by focusing on the basic human tendencies to approach/attract, avoid/repulse and synchronize/align. We believe that our model demonstrates the benefits that arise from combining biological models of animal behavior with insights from neuroscience and psychology, to improve our understanding of social behavior in humans. Interestingly, similar conclusions (namely, a failure of approach/avoidance) have been drawn from studying a range of neuropsychiatric disorders, from autism spectrum conditions (Pfaff and Barbas, 2019) to major depression (Ironside *et al*., 2020). However, perhaps the more intriguing implication is that the comorbidity of loneliness and other disorders (major depression and anxiety) might arise from a common pathophysiological mechanism that can be explained in terms of herding. Furthermore, while our model focuses on the four brain systems of herding, other systems are also implicated in loneliness. Specifically, alterations related to loneliness were found in cognitive control networks (Wong *et al*., 2022), attentional networks (Tian *et al*., 2014, 2017), the default mode network (Lam *et al*., 2021), the visual network in response to social threat (Cacioppo and Hawkley, 2009) and its connectivity with affective networks (Tian *et al*., 2017). These networks are likely to interact with the herding systems to regulate social behavior.

Our synthesis can be applied to guide healthcare providers to understand why loneliness develops. Critically, considering that challenges in herding dynamics may contribute to the persistence of loneliness, interventions targeting the three components of herding—attraction, repulsion and alignment—hold the potential to design effective strategies for reducing loneliness. Research shows that movement synchrony reduces feelings of loneliness such as in group dancing (Humphries *et al*., 2023), and synchronized movement in older adults showed to reduce loneliness and to increase oxytocin (Elheja *et al*., 2021). A recent review suggests that dance movement therapy is effective in reducing the severity of symptoms common in depression, autism, schizophrenia and somatoform disorder (Millman *et al*., 2021), which are correlated with higher rates of loneliness (Ludwig *et al*., 2020; Haigh and Walford, 2021). Likewise, a recent study demonstrated that exposing participants to physical contact was associated with reduced levels of loneliness (Heatley Tejada *et al*., 2020). Notably, studies show that synchronization is trainable (Su and Keller, 2020), pointing to neuroplasticity of herding-related networks. Therefore, interventions that emphasize promoting physical proximity (e.g. physical touch) and alignment (e.g. group dances or singing) may be particularly effective for the attenuation of loneliness (Millman *et al.*, 2021; Heatley Tejada *et al*., 2020).

To conclude, we propose that this framework should be investigated as a possible overarching explanation that may account for the bigger part of the variance that we witness in loneliness and social behavior studies. However, the current review is a theoretical model that has yet to be tested directly. We therefore call researchers to examine our hypothesis in empirical studies and approve, dispute or refine our model. If correct, the herding model of loneliness can inform interventions aiming at cultivating lonely individuals’ capacity to approach, avoid and align in an attuned fashion may contribute to improvement in how they respond to social interactions, resulting in enhanced sense of connectedness and diminished loneliness.

## Data Availability

No new data were generated or analysed in support of this research.
